# Beyond the Cardiovascular Effects of Glucagon-like Peptide-1 Receptor Agonists: Body Slimming and Plaque Stabilization. Are New Statins Born?

**DOI:** 10.3390/biom13121695

**Published:** 2023-11-23

**Authors:** Dalgisio Lecis, Francesca Romana Prandi, Lucy Barone, Martina Belli, Domenico Sergi, Susanna Longo, Saverio Muscoli, Francesco Romeo, Massimo Federici, Stamatios Lerakis, Francesco Barillà

**Affiliations:** 1 Division of Cardiology, Department of Systems Medicine, Tor Vergata University, 00133 Rome, Italy; francescaromanaprandi@gmail.com (F.R.P.); lucy.barone82@gmail.com (L.B.); martynebelli@gmail.com (M.B.); domenicosergi@gmail.com (D.S.); saveriomuscoli@gmail.com (S.M.); francesco.barilla@ptvonline.it (F.B.); 2 Division of Cardiology, Mount Sinai Hospital, Icahn School of Medicine at Mount Sinai, 1 Gustave L. Levy Place, New York, NY 10029, USA; stamatios.lerakis@mountsinai.org; 3 Cardiovascular Imaging Unit, San Raffaele Scientific Institute, 20132 Milan, Italy; 4 Department of Systems Medicine, Tor Vergata University, 00133 Rome, Italy; susanna.longo@uniroma2.it (S.L.); federicm@uniroma2.it (M.F.); 5 Faculty of Medicine, UniCamillus-Saint Camillus International University of Health and Medical Sciences, 00131 Rome, Italy; romeocerabino@gmail.com

**Keywords:** GLP-1RAs, diabetes mellitus, cardiovascular disease, prevention, atherosclerosis

## Abstract

Atherosclerosis is a chronic inflammatory disease characterized by lipid and inflammatory cell deposits in the inner layer of large- and medium-sized elastic and muscular arteries. Diabetes mellitus (DM) significantly increases the risk of cardiovascular diseases and the overall and cardiovascular mortality, and it is a pro-atherogenic factor that induces atherosclerosis development and/or accelerates its progression through a multifactorial process. Glucagon-like peptide-1 receptor agonists (GLP-1RAs) are a new class of drugs, belonging to the armamentarium to fight type 2 DM, that have shown robust reductions in atherosclerotic events and all-cause mortality in all studies. Preclinical studies have shown that GLP-1RAs play a role in the immunomodulation of atherosclerosis, affecting multiple pathways involved in plaque development and progression. In this review, we wanted to explore the translational power of such preclinical studies by analyzing the most recent clinical trials investigating the atheroprotective effect of GLP-1RAs.

## 1. Introduction

Atherosclerosis is a chronic inflammatory disease characterized by lipid and inflammatory cell deposits in the inner layer of large- and medium-sized elastic and muscular arteries. In 1913, Nikolai N. Anichkov was the first to recognize the role of cholesterol in the development of atherosclerosis (“the lipid hypothesis”) through a pioneering work that demonstrated the creation of fatty lesions in the arteries of cholesterol-fed rabbits [1]. Anichkov was also the first to describe the “cholesterinesterphagozyten” derived from macrophages that are known today as foam cells. In 1951, Gofman et al. used the newly invented ultracentrifuge to characterize human blood lipids and lipoproteins [2]. Later, they observed a correlation between high low-density lipoprotein (LDL) levels, low high-density lipoprotein levels, and heart attacks [3], developing an “atherogenic index” to estimate the risk of coronary artery disease [4]. In 1973, Brown and Goldstein discovered the LDL receptor and described its key role in the regulation of cholesterol homeostasis [5]. LDLs are the most numerous of the ApoB-containing lipoproteins, and they represent the principal carrier of cholesterol in human plasma and are a key deliverer of cholesterol to the artery wall.

However, atherosclerosis does not result simply from a passive accumulation of lipids within the artery wall but is an inflammatory disease [6,7]. Inflammatory processes contribute to all phases of the life cycle of atherosclerotic plaques, from initiation to the ultimate thrombotic complications [8].

The initial stages of human atherogenesis are characterized by LDL transcytosis and efflux across a compromised endothelium having high permeability, with LDL accumulation in the subendothelium [9]. The binding of LDL to the arterial intima matrix proteoglycans leads to LDL focal retention, with increased oxidative modifications since the vascular wall may be devoid of efficient antioxidant mechanisms [10]. Oxidized-LDL (ox-LDL) retained in the arterial wall represents the initial “injury” that, through interaction with the endothelial lectin-type ox-LDL receptor 1 (LOX-1), activates the inflammatory “response to injury” with the involvement of innate and adaptive immunity.

The interaction between ox-LDL and LOX-1 influences multiple cell types, such as endothelial cells, monocytes, macrophages, vascular smooth muscle cells, fibroblasts, and platelets. Therefore, ox-LDLs through LOX-1 are responsible for several downstream effects, including endothelial activation with increased expression of adhesion molecules, monocyte recruitment with macrophage differentiation and foam cell formation, apoptosis, smooth muscle cell proliferation and migration, platelet activation, extracellular matrix deposition, and fibroatheroma formation [11]. LOX-1 is also involved in the activation of the nucleotide-binding oligomerization domain-like receptor pyrin domain containing (NLRP3) inflammasome and in the increased expression of the angiotensin converting enzyme [12].

Inflammation and inflammatory cells are also involved in the acute complications of atherosclerosis. In addition to reducing the cholesterol content of plaque, lipid lowering reduces the accumulation of macrophages, which produce proteolytic enzymes, such as matrix metalloproteinases (MMPs, especially MMP-1, MMP-2, MMP-3, MMP-9, and MMP-13) [13]. MMPs degrade the collagen component of the fibrous cap, conferring plaque instability.

Several epidemiological studies, Mendelian randomization studies, and randomized controlled trials demonstrated a log-linear relationship between the absolute changes in plasma LDL and the risk of atherosclerotic cardiovascular diseases (ASCVDs) [14,15], which include coronary heart disease, cerebrovascular disease, peripheral artery disease, and aortic atherosclerotic disease. Mendelian randomization studies also showed that long-term exposure to lower LDL levels is associated with a lower risk of ASCVDs compared with shorter-term exposure to lower LDLs [16]. Therefore, LDL particles have both a causal and a cumulative effect on the risk of ASCVDs [14].

One of the cornerstones in therapy for the prevention of acute cardiovascular events in patients with high plasma LDL-c is statins. This ancient class of drugs has over time proven that, in addition to having a hypolipidemic effect, it reduces cardiovascular events through the contribution of pleiotropic atherosclerotic plaque-stabilizing effects. The JUPITER and HOPE-3 trials showed how the administration of statins in primary prevention reduces the occurrence of major adverse cardiac events (MACEs) compared to placebo in patients with a moderate cardiovascular risk [17,18].

One of the most important cardiovascular risk factors is diabetes mellitus (DM). Diabetes mellitus is a glucose metabolism disorder characterized by chronic hyperglycemia resulting from a deficit in insulin production and/or action. DM significantly increases the risk of cardiovascular diseases (CVDs), overall mortality, and cardiovascular mortality, with CVD accounting for about two-thirds of the overall deaths in patients with type 2 DM (T2DM). DM is a pro-atherogenic factor that induces atherosclerosis development and/or accelerates its progression through a multifactorial process. Patients with DM present a higher prevalence of most of the reversible classic risk factors for atherosclerosis, such as hypertension, obesity, and dyslipidemia [19], but this only partially explains the excess coronary heart risk in T2DM [20]. Metabolic alterations, such as chronic hyperglycemia, insulin resistance, and increased metabolism of free fatty acids, result in oxidative stress, inflammation, advanced glycation end product formation, and abnormalities in calcium homeostasis, leading to apoptosis, fibrosis, and endothelial dysfunction [21], which represents the main initiating factor in the pathogenesis of diabetic vascular complications. Other factors are the influence of diabetic cardiomyopathy (DM-induced cardiac remodeling resulting in diastolic and/or systolic dysfunction) [21], genetic and epigenetic modifications [22], and the possible adverse effect of hypoglycemic agents.

Recently a new class of drugs, the glucagon-like peptide-1 receptor agonists (GLP-1RAs), gained attention and relevance in the treatment of T2DM because of their beneficial effects in MACE reduction.

## 2. GLP-1 Receptors and Their Agonists

Glucagon-like peptide-1 (GLP-1) is a 30-amino-acid peptide derived from preproglucagon and expressed predominantly in the gut, pancreas, and brain [23,24]. In the gut, the two bioactive forms of GLP-1, GLP-17e37 and GLP-17e36 amide, are secreted by epithelial enteroendocrine L cells in response to an oral glucose load. Furthermore, the intestinal release of GLP-1 is stimulated by the gut microbiota, which use undigested food nutrients, fiber, and bile acids to produce metabolites and induce further secretion of GLP-1 from enteroendocrine L cells [24]. The half-life of native GLP-1 is only ~2–3 min. It is degraded locally by the enzyme dipeptidyl peptidase-4 (DPP4) and minimally by the neutral endopeptidase, and it is eliminated by the kidney. Therefore, only 10–15% of endogenous GLP-1 reaches the systemic circulation [23,25].

GLP-1 receptors (GLP-1Rs) are coupled to G proteins, which stimulate the production of cyclic adenosine monophosphate, and they are ubiquitously located. In the pancreas, GLP-1 mediates the insulin secretory response to glucose ingestion with the direct activation of GLP-1Rs on beta cells and through paracrine mechanisms involving vagal afferents. GLP-1 also inhibits glucagon secretion and endogenous glucose production by acting directly on pancreatic alpha cells or by stimulating somatostatin production from delta cells. The final effect is to decrease the postmeal hyperglycemia. Moreover, GLP-1Rs are expressed in the hindbrain, hypothalamus, hippocampus, and mesolimbic system. At this site GLP-1 suppresses the appetite and inhibits gastric emptying, promoting weight loss and reducing postprandial hyperglycemia. The inhibitory effect on appetite also depends on the paracrine action of GLP-1 via intestinal vagal afferents that transmit the signal to the hypothalamus [24,26,27]. GLP-1Rs are also localized on white and brown adipose tissue where they increase energy expenditure. They have lower expression in the heart, lung, intestine, muscle, kidney, liver, and peripheral nervous system [23,24,25,28].

All available GLP-1 RAs are synthetic peptides derived from human GLP-1 or exendin-4, a 39-amino-acid peptide obtained from the saliva of the lizard *Heloderma awareum*. GLP-1RAs are more resistant to degradation than native GLP-1 [29]. Based on their pharmacokinetic profile, they are divided into short-acting agonists (exenatide and lixisenatide) and long-acting agonists (liraglutide; long-acting exenatide; albiglutide; dulaglutide; and long-acting, subcutaneous or oral semaglutide) [26,30]. Short-acting GLP-1RAs have a short half-life and are administered before a meal once or twice a day, showing higher postprandial plasma concentrations than in the fasting. Therefore, they reduce postprandial hyperglycemia and influence gastrointestinal motility. Vice versa, long-acting GLP-1RAs are administered once a week, and they reduce fasting blood glucose predominantly through direct pancreatic action, have greater body weight reduction potential, and show fewer effects on gastrointestinal motility [23]. GLP-1RAs also show nonglycemic effects, including decreases in weight; cytoprotective and proliferative effects on alpha, beta, and delta cells; and improvements in systolic blood pressure and cardiovascular risk markers, such as triglycerides and total cholesterol [24,26]. Their effect is glucose dependent, so the risk of hypoglycemia is very low [30]. The most observed adverse effect is intestinal nausea, which could be reduced by using low doses at the beginning of therapy [26].

The administration of GLP-1RAs can be both subcutaneous and oral. The main limitations in their oral administration are the poor absorption through the gastrointestinal tract and the rapid degradation caused by proteolytic enzymes and the acidic environment of the stomach. Oral semaglutide approved by the FDA in 2019 is coformulated with sodium N-[8-(2-hydroxybenzoyl) aminocaprylate] (SNAC). The hydrophobic property of SNAC increases the lipophilicity of semaglutide, facilitating its transcellular absorption across the gastric epithelium. Subsequently, the molecule undergoes cleavage of the noncovalent bond between semaglutide and SNAC, with a release of semaglutide into the circulation [23,31].

## 3. GLP-1RAs and Reduction in Cardiovascular Risk

CVDs are the leading cause of morbidity and mortality in patients with diabetes; therefore, the prevention of cardiovascular (CV) complications is essential in the treatment of T2DM [32]. In recent years, there has been an expansion of the armamentarium for T2DM treatment with drugs that are not limited exclusively to glycemic control but also have positive effects on reducing CV risk. Among these, sodium-glucose co-transporter 2 inhibitors (SGLT2-Is) and GLP-1RAs proved to have several vascular actions going beyond simple antihyperglycemic effects. GLP-1RAs are a new class of drugs that showed robust reductions in atherosclerotic events and all-cause mortality in all studies, with the exception of lixisenatide [33]. The LEADER (Liraglutide and Cardiovascular Outcomes in Type 2 Diabetes) is the first trial to demonstrate positive outcomes in CVDs in patients with T2DM [34]. Liraglutide resulted in a statistically significant reduction not only in the incidence of MACEs but also in CV and all-cause mortality. However, no differences were observed in rates of nonfatal myocardial infarction, nonfatal stroke, or hospitalization for heart failure between the liraglutide and placebo arms. Husain et al. performed an analysis to assess the effects of oral and injectable semaglutide versus placebo on MACEs in subjects with T2DM in a combined population from SUSTAIN 6 and PIONEER 6 [35]. The abovementioned analysis showed a significant protection against MACEs, equal to 24%, in the semaglutide group compared with the control group [35]. These results [36] are particularly significant considering the small sample size and the brief duration of observation [37]. Most CV outcome trials with GLP-1RAs recruited patients with T2DM characterized by established CVD or with a high risk of CV events. Indeed, these studies were originally designed primarily as safety studies, and the accumulation of a large number of CV events in high-risk patients was a strategy to limit the sample size and duration of these trials [37]. In the EXSCEL (Effects of Once-Weekly Exenatide on Cardiovascular Outcomes in Type 2 Diabetes) trial [38], exenatide failed to demonstrate superiority in preventing CV events, despite having a 9% reduction in the incidence of the primary endpoint (first occurrence of death from cardiovascular causes, nonfatal MI, or nonfatal stroke). The lack of cardiovascular efficacy in the EXSCEL trial may be related to multiple factors. The median follow-up time in the EXSCEL trial was shorter than that in the LEADER trial (3.2 years vs. 3.8 years), as was the duration of exposure to the trial regimen (2.4 years vs. 3.5 years); in addition, the baseline glycated hemoglobin level in the EXSCEL trial was lower than that in the LEADER trial (8.0% vs. 8.7%), and the rate of discontinuation of the trial regimen was higher. AMPLITUDE-O (Cardiovascular and Renal Outcomes with Efpeglenatide in Type 2 Diabetes), on the other hand, is a trial in which weekly subcutaneous injections of efpeglenatide (4 or 6 mg) for a median of 1.8 years led to a 27% lower risk of incident MACEs and a 32% lower risk of a composite renal outcome event than placebo among persons with T2DM and either a history of CVD or current kidney disease [39]. A meta-analysis summarized the results of these randomized trials, showing a significant reduction in MACEs, CV death, stroke, myocardial infarction, death from all causes, heart failure, and kidney disease, emphasizing the important glycometabolic effects and extraglycemic actions that would determine a significant clinical benefit in patients with T2DM [40]. In Table 1, all the principal trials investigating the CV outcomes upon GLP-1RA administration are listed.

The GLP-1RAs were shown to have pleiotropic CV effects [41] including reduction in blood pressure (BP) [42]. Specifically, the use of GLP-1RAs results in a reduction in BP (reduction in systolic BP by 1 mmHg with lixisenatide and by 5.4 mmHg with semaglutide) through a natriuretic and vasodilatory effect, likely mediated by the atrial natriuretic peptide, released as a result of binding to specific receptors expressed in the atria [43]. This beneficial effect on blood pressure reduction exerted by GLP-1RAs is consistent with the reduction in CV risk associated with their use. Endothelial cells play a key role in BP control. GLP-1RAs stimulate acetylcholine-induced vasodilation and promote nitric oxide release, thus reducing vascular tone with benefits to both systolic and diastolic BP [44]. A recent study demonstrated that acute pharmacological stimulation of GLP-1R increases eGFR by promoting diuresis and natriuresis through the inhibition of the major renal proximal tubule sodium reabsorption pathway. In particular, Skov et al. showed that GLP-1 infusion declined angiotensin II (ATII) and induced natriuresis in healthy young men [45]. Le et al. hypothesized that GLP-1R activation through exendin-4 (Ex 4) declined intrarenal RAS activity, leading to a reduction in the activity of the ATII-mediated TGF-β1/Smad3 signaling pathway in mice [46]. Although there are now several pieces of evidence on the benefits of GLP-1RAs to the kidney and BP control, further studies are needed to evaluate the combination of these drugs with antihypertensive agents, especially in patients with high cardiometabolic risk [47].

## 4. Metabolic Effects of GLP-1RA Administration

The administration of GLP-1RAs, like liraglutide, exenatide, semaglutide, and lixisenatide, has multiple metabolic effects, such as glucose-dependent insulin secretion, slower gastric emptying, increased natriuresis and diuresis, increased pancreatic β-cell mass, regulation of lipid metabolism, and decreased fat deposition, and has attracted considerable interest in recent years for its role in improving glycemic control and aiding weight loss in T2DM patients [48].

Drugs like liraglutide not only enhance pancreatic β-cell mass, leading to better oral glucose tolerance and reduced peak serum glucose timings, but also contribute to weight management by lowering body weight, HbA1c, and liver fat content [49]. Exenatide, semaglutide, and lixisenatide have shown efficacy in controlling postprandial glucose levels, an important aspect of diabetes management [44,45,46,47].

Remarkably, these agents also address dyslipidemia, a common comorbidity in T2DM. A significant meta-analysis highlighted GLP-1RAs’ ability to lower total cholesterol, low-density lipoprotein cholesterol (LDL-C), and triglyceride levels compared to other antidiabetic agents or placebo. In particular, liraglutide and taspoglutide stand out for their cholesterol-lowering effects, notably impacting total cholesterol and triglycerides, showcasing a broader therapeutic impact beyond glucose modulation [50].

This lipid-modifying potential is not confined to diabetic patients alone; individuals with obesity also experience reductions in the numbers of small- and medium-sized LDL particles and the total cholesterol with GLP-1RA treatment [51]. As such, the utility of GLP-1RAs might expand to the improvement of cardiovascular risk profiles, given that dyslipidemia is a key player in cardiovascular disease.

In sum, the metabolic effects of GLP-1RAs encapsulate a wider approach to T2DM treatment, addressing various aspects of the disorder from glycemic control to weight reduction and lipid profile improvement. These effects are possibly mediated through mechanisms that include, but are not limited to, improved glycemic control, reduced hepatic lipid production, enhanced fatty acid oxidation, and upregulated LDL receptor activity [52], offering a multifaceted therapeutic strategy for both diabetes and its associated metabolic disturbances.

## 5. Atherosclerotic Plaque Pathways Targeted by GLP-1RAs

The use of liraglutide and semaglutide, as shown in the LEADER and SUSTAIN-6 trials, reduces the occurrence of MACEs in high-risk CVD patients with diabetes. This effect is the consequence of GLP-1RAs’ anti-atherosclerotic action.

Some preclinical studies investigated how different pathways involved in atherosclerosis initiation and development are affected by the administration of GLP-1RAs. The genesis of foam cells is critical for the development of the atherosclerotic plaques. Acetyl-coenzyme A acetyltransferase 1 (ACAT1) expression is upregulated during the differentiation of monocytes to macrophages, and it is abundantly expressed in macrophage foam cells of atherosclerotic lesions [53]. It has been shown that, during ischemic stress, macrophages increase their uptake of ox-LDL cholesterol, leading to increased ACAT1 activity and the formation of toxic levels of cholesterol esters [54]. The esterification of cholesterol results in its storage in cellular lipid droplets, making it unavailable for ATP-binding cassette A1–mediated efflux from the cell and leading to the formation of macrophage foam cells. The treatment of mouse peritoneal macrophages with an ACAT inhibitor significantly reduced 7-ketocholesterol-induced apoptosis [55]. Kharbanda et al. were the first to report the effects of systemic ACAT inhibition in humans. Hypercholesterolemic human subjects treated for 8 weeks with avasimibe, an ACAT inhibitor, showed a small reduction in plasma cholesterol levels but had significantly lower levels of circulating tumor necrosis factor-α (TNF-α), a pro-inflammatory cytokine [56]. Tashiro et al. showed that liraglutide could prevent the development of atherosclerotic lesions by suppressing macrophage foam cell formation mainly associated with ACAT1 downregulation [57].

Nagashima et al. showed that the infusion of native incretins (GLP-1 and GIP) in ApoE^−/−^ mice also resulted in a reduction in foam cell formation through the downregulation of the CD36 and ACAT1 pathways [58]. GLP-1 and GIP receptors were both detected in Apoe^−/−^ mouse macrophages. CD36 plays a significant role in the pathogenesis of atherosclerosis by serving as a highly specific receptor for oxidized phospholipids prevalent in ox-LDL. The interaction of ox-LDL with CD36 triggers a signaling cascade that is necessary for ox-LDL uptake and foam cell formation and that alters cytoskeletal dynamics and inhibits migration, thus contributing to the trapping of foam cells within the atherosclerotic plaque [59].

Rapikovski et al. showed the effects of semaglutide on aortic plaque formation in ApoE^−/−^ and LDLr^−/−^ mice through the evaluation of gene expression from collected aorta samples. The expression of 275 genes involved in the atherosclerotic process has been investigated [60]. This interesting and comprehensive analysis documented that the administration of a daily dose of semaglutide of 60 μg/kg for 12 to 14 weeks in ApoE^−/−^ mice or for 17 weeks in LDLr^−/−^ mice impacted multiple pathways and stages characterizing the pathogenesis of atherosclerosis. Semaglutide reduced the leukocyte recruitment by the downregulation of IL-6, IL-1RN, and chemokine (C-C motif) ligand 2 (CCL2) gene expression. Moreover, the leukocyte rolling, adhesion, and extravasation process was affected by E-selectin (SELE) and vascular cell adhesion molecule 1 (VCAM-1) downregulation. Interestingly, semaglutide probably plays a role in plaque vulnerability, since the gene expression of proteins involved in extracellular matrix turnover (MMP-3 and MMP-13) and plaque hemorrhage (CD163) was reduced in the semaglutide-treated group [60].

The anti-inflammatory effects of semaglutide were investigated by analyzing the plasma levels of the inflammatory cytokines TNF-α and IFN-γ in lean C57BL/6J mice challenged with a single dose of LPS. Pretreatment with semaglutide reduced the TNF-α and IFN-γ response after LPS exposure [60]. An important pro-inflammatory cytokine that also plays a role in immune cell recruitment is osteopontin (OPN) [61]. Giachelli et al. demonstrated that elevated circulating OPN levels were associated with an increased CVD risk in T2DM [62]. Semaglutide decreased plasma OPN levels following an LPS challenge; moreover, OPN expression was decreased in aortic tissue with semaglutide treatment [60].

Lastly, liraglutide has been shown to be able to affect the M1/M2 ratio, promoting an M2 phenotype in apoE^−/−^ mice with an atheroprotective effect [63]. Bruen et al. demonstrated that liraglutide reduced pro-inflammatory immune cell populations and mediators from plaque-burdened murine aortas in vivo and augmented pro-resolving bone-marrow-derived macrophages in the attenuation of atherosclerotic disease [64]. A recent study compared the distribution of macrophages and their surface GLP-1Rs in patients with confirmed or unconfirmed CVD through coronary angiography and without bias from the difference in T2DM distribution or GHbA1c level between the two groups [65]. The data show that the expression of GLP-1Rs on total and M2 macrophages differed between the CVD group and the healthy control group (*p* < 0.05). The expression of GLP-1Rs was higher in the healthy group. The two groups did not exhibit any notable distinctions in the surface expression of GLP-1Rs on M1 macrophages or the proportions of total, M1, and M2 macrophages. This implies that alterations in GLP-1R expression levels are a more responsive indicator than the proportional macrophage composition when considering the chronic inflammatory progression of atherosclerosis. Specifically, the diminished presence of GLP-1Rs on M2-type anti-inflammatory macrophages in individuals with coronary heart disease (CHD) could elucidate why GLP-1RAs offer therapeutic benefits for CHD that are not contingent on glycemic control.

**Figure 1:**
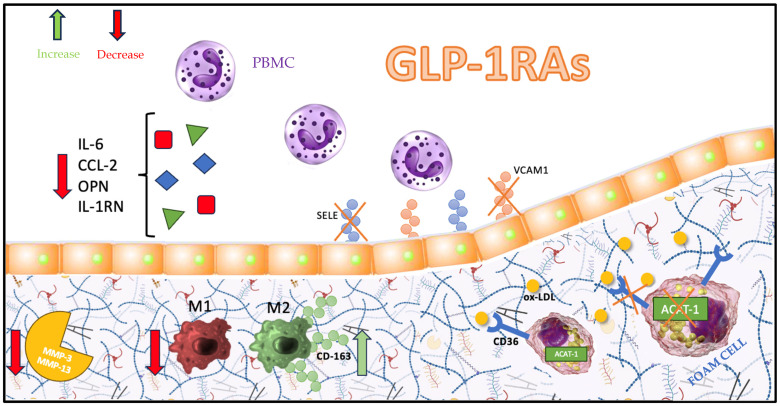
Pathways involved in plaque development and progression affected by GLP-1RAs. Chemokine C–C motif ligand 2 (CCL2), cholesterol acyltransferase 1 (ACAT1), cluster of differentiation 36 molecule (CD36), cluster of differentiation 163 molecule (CD163), E-selectin (SELE), interleukin-1 receptor antagonist (IL-1RN), interleukin-6 (IL-6), macrophage phenotype 1 (M1), macrophage phenotype 2 (M2), metalloproteinase-3 (MMP-3), metalloproteinase-13 (MMP-13), osteopontin (OPN), oxidized-LDL (ox-LDL), peripheral blood mononuclear cell (PBMC), vascular cell adhesion molecule 1 (VCAM-1).

Hence, GLP-1R potentially serves as a mediator in the modulation of macrophage polarization and in the release of distinct inflammatory molecules via the NLRP3/Caspase1 pathway, thereby influencing the progression of atherosclerosis [66]. The favorable inhibitory outcomes of GLP-1RAs on both the occurrence and progression of cardiovascular diseases, along with their underlying mechanisms, may not be exclusively contingent upon their use in treating T2DM. GLP-1RAs operate independently of T2DM-induced hypoglycemic effects and positively impact the cardiovascular system [67]. Furthermore, GLP-1R likely modulates macrophage polarization toward the M2 phenotype, thereby contributing to its protective function in advancing coronary atherosclerosis [60]. The role of GLP-1RAs in the immunomodulation of atherosclerosis is depicted in Figure 1.

## 6. Clinical Implications

GLP-1RAs were initially approved for the treatment of T2DM. Among the GLP-1RAs, liraglutide has been approved for the treatment of obesity as well; therefore, it becomes reasonable to wonder whether the administration of these drugs to other categories of patients such as nondiabetic patients or high-risk cardiovascular patients could be effective and safe to reduce cardiovascular events.

The STEP 1–5 trials demonstrated that adults without diabetes who were overweight or obese had clinically relevant weight loss with weekly injections of semaglutide (2.4 mg) added to lifestyle changes [68,69]. Moreover, the analysis by Amaro et al. showed an improvement in cardiometabolic risk factors [68], but it did not focus on CV outcomes.

Liao et al. demonstrated that GLP-1RAs can drastically lower visceral fat and hepatic fat content in adults. Their study demonstrated that GLP-1RAs can reduce visceral fat and hepatic fat content in patients with both T2DM and nonalcoholic fatty liver disease, suggesting that GLP-1RAs should be more widely used in the treatment of these conditions. Thus, GLP-1RAs could be recommended as an alternative treatment for various metabolic diseases that cause visceral fat accumulation [70].

The ongoing SELECT study is evaluating insights into the intersection of obesity, diabetes, and CVD. In that study, which is one of the largest CV outcome trials in the field of obesity, semaglutide at a dose of 2.4 mg once weekly is being evaluated to reduce CV events in people with overweight or obesity and previous CVD but without diabetes [71].

Aside from the reduction in CV risk caused by GLP-1RAs, in recent years, there has been growing interest in the potential effects of GLP-1RAs in the management and prevention of various neurodegenerative diseases, including Alzheimer’s disease [72]. Several preclinical and animal studies have suggested that GLP-1RAs may have neurotrophic and neuroprotective effects, by reducing neuroinflammation, oxidative stress, apoptosis, and mitochondrial dysfunction and by promoting the growth and repair of neural cells, potentially enhancing cognitive functions [73,74,75]. GLP-1RAs may also have a direct impact by reducing the levels of beta-amyloid and tau hyperphosphorylation in Alzheimer’s disease [76]. In addition, neuroprotective effects may also be exerted through an indirect mechanism; it is known that T2DM shares common pathophysiological mechanisms with the development of cognitive disorders related to insulin resistance. GLP-1RAs can improve glucose metabolism and increase insulin sensitivity in neurons [77]. These findings point to the consideration of GLP-1RAs as a promising treatment option for neurodegenerative diseases. Data from human clinical trials have shown an improvement in some brain markers but have not thus far demonstrated a strong correlation to cognitive scores [75,78]. Therefore, more data from ongoing trials are needed to determine safety and efficacy in the prevention and management of neurodegenerative disorders such as Alzheimer’s and Parkinson’s diseases.

## 7. Conclusions and Future Directions

In recent years, multiple studies on CVDs have demonstrated the effectiveness of GLP-1RAs in preventing cardiovascular events, including acute myocardial infarction and stroke, and reducing related mortality, including all-cause mortality.

GLP-1RAs appear to exert a specific influence on cardiovascular outcomes by mitigating endothelial dysfunction and oxidative stress. Additionally, these agents may influence the advancement of atherosclerosis through their anti-inflammatory impact on endothelial cells, their capacity to diminish smooth muscle cell proliferation, and their ability to attenuate the pro-inflammatory responses of macrophages [67].

This “plaque stabilization” effect exerted by GLP-1RAs resembles the pleiotropic effects of statins, which were approved a long time ago as first-line therapy to lower blood cholesterol levels. The JUPITER and LIPID trials showed how the administration of statins in primary and secondary prevention, respectively, were effective in reducing MACEs [18,79]. In that circumstance, researchers discovered that statins induced a “side” effect resulting in a reduction in acute cardiovascular events and plaque stabilization. By transposing the experience derived from the administration of GLP-1RAs to T2DM patients in terms of MACE reduction, it becomes reasonable to suppose that the administration of such drugs to nondiabetic high-risk cardiovascular patients with established atherosclerotic cardiovascular disease could be beneficial. This assertion can be considered valid since GLP-1RAs’ effect is glucose dependent, so the risk of hypoglycemia is very low in nondiabetic patients as well. Clinical trials with a wider range of participants may reveal differences that can elucidate this phenomenon, and further research is needed to determine the specific mechanisms and causal relationships.

## Figures and Tables

**Table 1:** Key results from cardiovascular outcome trials.

Trial (Duration)	GLP-1RA	Primary Endpoint	Results
ELIXA (5 years)	Lixisenatide	CV death, MI, stroke, or hospitalization for UA	No benefit
LEADER (1.5 years)	Liraglutide	First occurrence of death from CV causes, nonfatal MI, or nonfatal stroke	Significant decrease
SUSTAIN-6 (2.1 years)	Injectable semaglutide	First occurrence of CV death, nonfatal MI, or nonfatal stroke	Significant decrease
PIONEER 6 (15.9 months)	Oral semaglutide	First occurrence of death from CV causes, nonfatal MI, or nonfatal stroke	No benefit
EXSCEL (7 years)	Exenatide	First occurrence of death from CV causes, nonfatal MI, or nonfatal stroke	No benefit
HARMONY (2.4 years)	Albiglutide	CV death, nonfatal MI, or stroke	Significant decrease
REWIND (8.4 years)	Dulaglutide	First occurrence nonfatal MI, nonfatal stroke, or death from CV causes	No benefit
AMPLITUDE-O (3 years)	Efpeglenatide	First MACE: composite of nonfatal MI, nonfatal stroke, or death from CV or undetermined causes	Significant decrease

CV: cardiovascular; MI: myocardial infarction; UA: unstable angina; MACE: major adverse cardiovascular event.
